# The Case for the Entourage Effect and Conventional Breeding of Clinical Cannabis: No “Strain,” No Gain

**DOI:** 10.3389/fpls.2018.01969

**Published:** 2019-01-09

**Authors:** Ethan B. Russo

**Affiliations:** International Cannabis and Cannabinoids Institute, Prague, Czechia

**Keywords:** cannabis, cannabinoid, marijuana, hemp, genomics, genetically modified organism, tetrahydrocannabinol, cannabidiol

## Abstract

The topic of Cannabis curries controversy in every sphere of influence, whether politics, pharmacology, applied therapeutics or even botanical taxonomy. Debate as to the speciation of Cannabis, or a lack thereof, has swirled for more than 250 years. Because all Cannabis types are eminently capable of cross-breeding to produce fertile progeny, it is unlikely that any clear winner will emerge between the “lumpers” vs. “splitters” in this taxonomical debate. This is compounded by the profusion of Cannabis varieties available through the black market and even the developing legal market. While labeled “strains” in common parlance, this term is acceptable with respect to bacteria and viruses, but not among Plantae. Given that such factors as plant height and leaflet width do not distinguish one Cannabis plant from another and similar difficulties in defining terms in Cannabis, the only reasonable solution is to characterize them by their biochemical/pharmacological characteristics. Thus, it is best to refer to Cannabis types as chemical varieties, or “chemovars.” The current wave of excitement in Cannabis commerce has translated into a flurry of research on alternative sources, particularly yeasts, and complex systems for laboratory production have emerged, but these presuppose that single compounds are a desirable goal. Rather, the case for Cannabis synergy via the “entourage effect” is currently sufficiently strong as to suggest that one molecule is unlikely to match the therapeutic and even industrial potential of Cannabis itself as a phytochemical factory. The astounding plasticity of the Cannabis genome additionally obviates the need for genetic modification techniques.

## Introduction: Defining Terms

Earlier data on taxonomy of Cannabis was previously reviewed (Russo, 2007), which will be herein summarized and supplemented. Cannabis is a dioecious annual of the Cannabaceae family which traditionally includes hops, *Humulus* spp. Alternatively, *Cannabis* has also been assigned to Moraceae, Urticaceae, or even in the Celtidaceae families on the basis of chloroplast restriction site maps (Weigreffe et al., 1998), and chloroplast *mat* K gene sequences (Song et al., 2001). More recently, the Cannabaceae have subsumed eight genera: *Celetis, Pteroceltis, Aphananthe, Chaetachme, Gironniera, Lozanella, Trema*, and *Parasponia*, comprising 170 odd species (McPartland, 2018), a finding supported by genetic analysis of four plastid loci (Yang et al., 2013). Current research on fossil pollen samples associated with the ecological associations of *Cannabis* with steppe companion species (*Poaceae, Artemisia, Chenopodiaceae*), and *Humulus* (hops) with forest genera (*Alnus, Salix, Populus*), have established that although *Cannabis* seems to have originated in the Tibetan Plateau at least 19.6 million years ago, it has also been indigenous to Europe for at least a million years (McPartland et al., 2018), and refuted the conventional wisdom that this “camp follower” was brought there by man.

The species assignation of Cannabis itself is fraught with great debate. *Cannabis sativa*, meaning “cultivated Cannabis,” was so named by Fuchs, among others, in 1542 (Fuchs, 1999), an assignation 211 years before the systematization of botanical binomials Linnaeus in his *Species Plantarum* (Linnaeus, 1753*)*. Lamarck subsequently suggested *Cannabis indica*, a more diminutive intoxicating Indian plant from India, as a separate species (Lamarck, 1783). The issue has remained unresolved in the subsequent centuries with two opposing philosophies. Ernest Small has championed the single species concept (Small and Cronquist, 1976). Polytypic treatments of Cannabis also gained adherents (Schultes et al., 1974; Anderson, 1980) on morphological criteria suggesting separation of *Cannabis sativa* L. *Cannabis indica* Lam. and *Cannabis ruderalis* Jan., a scheme supported by systematic chemotaxonomy. Principal component analysis (PCA) of 157 Cannabis accessions from around the world assessed allozyme frequencies at 17 gene loci suggested a split (Hillig, 2005b). “S*ativa*” gene pools from eastern European ruderal samples were linked to narrow-leaflet European and Central Asian fiber and seed plants, while an “*indica”* grouping encompassed Far Eastern seed and fiber plants and drug plants with broad-leaflets from most of the rest of the world, along with wild accessions from the Indian subcontinent. Central Asian roadside samples (*Cannabis ruderalis)* were thought to represent a third group. Gas chromatography (GC) and starch-gel electrophoresis studies also suggested species separation of *sativa* and *indica* (Hillig and Mahlberg, 2004).

Agronomic factors in 69 samples suggested inclusion of eastern hemp and drug plants in *Cannabis indica* (Hillig, 2005a), a division supported by fragment length polymorphisms (Datwyler and Weiblen, 2006).

More recently, PCA seemed to point to terpenoid content as the most convincing distinguishing chemotaxonomic markers between putative *sativa* and *indica* species (Elzinga et al., 2015). Similarly, PCA was felt to separate drug Cannabis from hemp (Sawler et al., 2015). A recent study demonstrated demarcation of Cannabis drug from hemp accessions via genotyping of 13 microsatellite loci across the genome, not merely genes affecting cannabinoid or fiber production (Dufresnes et al., 2017). Professor Giovanni Appendino has reported the presence of the *cis-*Δ^9^-THC stereo-isomer only in the hemp accessions (Giovanni Appendino, personal communication). However, these distinctions may well pass by the wayside given the current trend to crossbreed hemp with drug cultivars to avoid legislative restrictions on THC content.

The Cannabis species controversy, *Cannabis sativa* vs. *indica* vs. *afghanica*, has continued unabated to the current day with impassioned arguments advanced by the protagonists (Clarke and Merlin, 2013, 2016; Small, 2015; McPartland and Guy, 2017; Small, 2017). This author, having been on every side of the issue at one time or another, has chosen to eschew the irreconcilable taxonomic debate as an unnecessary distraction (Piomelli and Russo, 2016), and rather emphasize that only biochemical and pharmacological distinctions between Cannabis accessions are relevant. In his recent seminal review, McPartland agreed, “Categorizing Cannabis as either ‘Sativa’ and ‘Indica’ has become an exercise in futility. Ubiquitous interbreeding and hybridization renders their distinction meaningless.” (McPartland, 2018) (p. 210).

An additional non-sensical nomenclature controversy pertains in common parlance to Cannabis “strains,” an appellation that is appropriate to bacteria and viruses, but not plants (Bailey and Bailey, 1976; Usher, 1996; Brickell et al., 2009), especially so with Cannabis where the chemical variety, abbreviated “chemovar” is the most appropriate appellation (Lewis et al., 2018).

## The Cannabis Genome and Alternative Host Biochemical Production

2011 was a landmark year for Cannabis genomics, as Medical Genomics and Nimbus Informatics issued an online report on the complete 400 million base-pair genomic sequence, which was shortly joined by a draft genome and transcriptome (van Bakel et al., 2011).

This development sparked prominent publicity and controversy as to what it might portend. Whereas, the human genome was analyzed some 20 years earlier, the implications for Cannabis were subject to great speculation.

**FIGURE 1:**
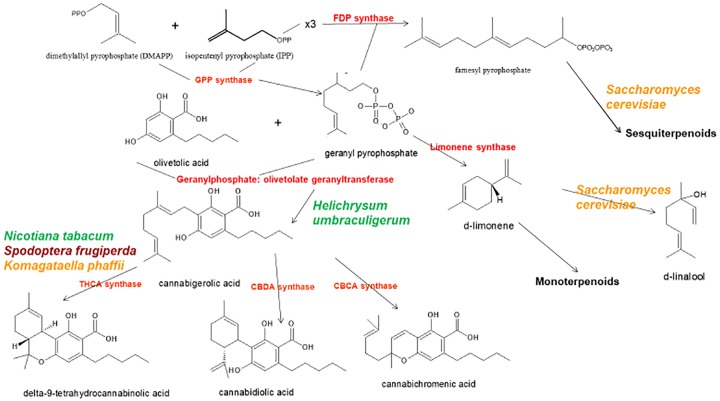
Biosynthetic pathways and enzymes (red) of *Cannabis sativa*, indication the natural species *Helichrysum umbraculigerum*, and alternative species (in color) that have been genetically modified to produce subsequent products [redrawn and updated from (Russo, 2011) using ACD/ChemSketch 2017.2.1].

The news catalyzed a flurry of new research, but considerable progress had already been achieved in applied Cannabis genetics. The identification and synthesis of Δ^9^-tetrahydrocannabinol (THC) was accomplished in Israel 1964 (Gaoni and Mechoulam, 1964), but it was not until much later before successful cloning of its biosynthetic enzyme, tetrahydrocannabinolic acid synthase (THCA synthase) (Sirikantaramas et al., 2004; Figure 1). Enzyme crystallization followed (Shoyama et al., 2005). Cannabidiolic acid synthase, which catalyzes cannabidiolic acid (CBDA), the precursor of cannabidiol (CBD), had been previously identified and produced in pure form (Taura et al., 1996; Figure 1). These developments stimulated additional findings, including the archeological phytochemical discovery of THCA synthase in a 2700 year old Cannabis cache from a tomb in Central Asia along with two previously unreported single nucleotide polymorphisms (SNPs) in the enzyme’s gene sequence (Russo et al., 2008).

By 2011, the enzymes for the production of the major phytocannabinoids had been identified. Similarly, selective advanced Mendelian breeding yielded Cannabis varieties rich in specific single components. Thus, high-THC and high-CBD plants were produced for pharmaceutical development (de Meijer et al., 2003; de Meijer, 2004), with analogous breeding of high-cannabigerol (CBG) (de Meijer and Hammond, 2005) and cannabichromene (CBC) lines (de Meijer et al., 2009a). The selective breeding also extended to propyl phytocannabinoid analogs, tetrahydrocannabivarin (THCV), cannabidivarin (CBDV), cannabigerivarin (CBGV), and cannabichromivarin (CBCV) (de Meijer, 2004). The availability of plants with high titers of these “minor cannabinoids” portend interesting new pharmaceutical applications (Russo, 2011; Russo and Marcu, 2017).

Access to the Cannabis genome might simplify production of THC-knockout plants via CRISPR technology (clustered regularly-interspaced short palindromic repeats). While this could be attractive for industrial hemp breeding, a prior generation of plant husbandry has already yielded hemp cultivars that easily fulfill international restrictions that require 0.1% or less THC content (Wirtshafter, 1997; McPartland et al., 2000; Small and Marcus, 2003). In fact, cannabinoid-free Cannabis with no functional cannabigerolic acid synthase (Figure 1) has also been produced conventionally (de Meijer et al., 2009b). Thus, it remains unclear that genetic engineering of Cannabis is even necessary for this plant whose incredible plasticity already displays bountiful biochemical diversity. Introduction of genetically modified organism (GMO) Cannabis would incite considerable controversy among certain segments of the population, and likely provoke a flurry of legal entanglements over patent and breeding rights.

One may easily imagine a variety of additional science fiction scenarios. In the 1990s an Internet hoax spread the rumor that an apocryphal Professor Nanofsky had introduced genes for THC production into oranges (*Citrus x. sinensis* (L.) Osbeck). Although this could be technologically achievable, such an effort would be no more than a laboratory carnival act in light of the prodigious cannabinoid production from Cannabis itself. A stealthy peppermint chemovar (*Mentha****x*** *piperita* Lamiaceae) sporting illicit phytocannabinoids in the glandular trichomes of its leaves might be more logical choice for such underground subversive daydreams and send rhizomes and runners along watercourses worldwide.

Prior claims of production of cannabidiol from hops (*Humulus lupulus* L. *Cannabaceae*) and flax (*Linum usitatissimum* L. *Linaceae*) are unsubstantiated, but cannabigerolic acid and cannabigerol were detected in South African *Helichrysum umbraculigerum* Less. *Asteraceae* (Bohlmann and Hoffmann, 1979; Appendino et al., 2015; Russo, 2016; Figure 1), but without reference to its concentration. This claim was confirmed recently with trace amounts observed from dried samples of aerial parts (Mark Lewis, personal communication).

Because the complexity of purely *de novo* biochemical synthesis of cannabinoids has been deemed non-cost effective (Carvalho et al., 2017), alternative microbial hosts have been suggested (Zirpel et al., 2017). In 2004, cDNA cloning of THCA synthase was achieved, allowing conversion of cannabigerolic acid (CBGA) to THC (Sirikantaramas et al., 2004), and an 8% THCA production in tobacco hairy roots (*Nicotiana tabacum* cv.Xanthi Solanaceae) was demonstrated on CBGA feeding (Figure 1). The enzyme was also expressed in the insect, *Spodoptera frugiperda* (J.E. Smith) *Noctuidae* (fall armyworm) via a recombinant baculovirus. Subsequently, this research group turned to yeasts, *Pichia pastoris* (now *Komagataella phaffii* Phaff *Saccharomycetaceae*) (Taura et al., 2007; Figure 1), and achieved a CBGA to THCA conversion of 98% over 24 h, with yield of 32.6 mg/L of medium. A recombinant form of THCA synthase proved 4.5X more efficient than in Cannabis and 12X that in *S. frugiperda.* This process was subsequently optimized with a 64.5-fold improvement in activity (Zirpel et al., 2018), with a reported production in *K. phaffii* of 3.05 g/L of THCA after 8 h of incubation at 37°C. A simple calculation provides that this yield could also be achieved from extraction of just 15 g of 20% THCA herbal Cannabis.

Cannabis terpenoid production is similarly possible in alternative hosts. *Saccharomyces cerevisiae* Meyen *ex* E.C. Hansen *Saccharomycetaceae* mutants deficient in farnesyl diphosphate synthase enzyme accumulate geranyl pyrophosphate instead, which is shunted into the production of medically useful terpenoid, linalool (Oswald et al., 2007; Figure 1). Similarly, other researchers have harnessed the biosynthetic capabilities of mitochondria in *S. cerevisiae* to increase farnesyl diphosphate production of sesquiterpenoids (Farhi et al., 2011), although not ones common to Cannabis.

At present, the existing Cannabis genomic sequences are not fully annotated. Consequently, applied foreknowledge and detective work will be necessary to acquire practical data on genetic function in Cannabis. The greatest potential in such investigation will lie in the realm of epigenetics, underlying hereditable changes in gene expression or phenotype of the plant. The most salient deficiency is a lack of knowledge regarding regulation of cannabinoid production. Understanding the biosynthetic pathways and regulation of terpene synthases producing the Cannabis terpenoids has barely been initiated (Booth et al., 2017) and remain ripe targets of additional research (Russo, 2011).

An additional problem in Cannabis husbandry remains a dearth of voucher specimens (which are prohibited by the US Drug Enforcement Administration without Schedule I license) and formal deposits of chemovar accessions in seed and tissue repositories. The latter has been accomplished by GW Pharmaceuticals, and independently by NaPro Research (Lewis et al., 2018) in the National Collection of Industrial, Food and Marine Bacteria (NCIMB) in Scotland. Many private companies have eschewed sharing germplasm due to legal restrictions and fear of loss of intellectual property.

## Cannabis Synergy

In 1998, Professors Raphael Mechoulam and Shimon Ben-Shabat posited that the endocannabinoid system demonstrated an “entourage effect” in which a variety of “inactive” metabolites and closely related molecules markedly increased the activity of the primary endogenous cannabinoids, anandamide and 2-arachidonoylglycerol (Ben-Shabat et al., 1998). They also postulated that this helped to explain how botanical drugs were often more efficacious than their isolated components (Mechoulam and Ben-Shabat, 1999). Although the single molecule synthesis remains the dominant model for pharmaceutical development (Bonn-Miller et al., 2018), the concept of botanical synergy has been amply demonstrated contemporaneously, invoking the pharmacological contributions of “minor cannabinoids” and Cannabis terpenoids to the plant’s overall pharmacological effect (McPartland and Pruitt, 1999; McPartland and Mediavilla, 2001; McPartland and Russo, 2001, 2014; Russo and McPartland, 2003; Wilkinson et al., 2003; Russo, 2011). Several pertinent examples of the entourage effect in Cannabis are illustrative:

In a randomized controlled trial of oromucosal Cannabis-based extracts in patients with intractable pain despite optimized opioid treatment, a THC-predominant extract failed to demarcate favorably from placebo, whereas a whole plant extract (nabiximols, *vide infra*) with both THC and cannabidiol (CBD) proved statistically significantly better than both (Johnson et al., 2010), the only salient difference being the presence of CBD in the latter.

In animal studies of analgesia, pure CBD produces a biphasic dose-response curve such that smaller doses reduce pain responses until a peak is reached, after which further increases in dose are ineffective. Interestingly, the application of a full spectrum Cannabis extract with equivalent doses of CBD eliminates the biphasic response in favor of a linear dose-response curve such that the botanical extract is analgesic at any dose with no observed ceiling effect (Gallily et al., 2014).

A recent study of several human breast cancer cell lines in culture and implanted tumors demonstrated superiority of a Cannabis extract treatment to pure THC, seemingly attributable in the former to the presence of small concentrations of cannabigerol (CBG) and tetrahydrocannabinolic acid (THCA) (Blasco-Benito et al., 2018).

Anticonvulsant effects of cannabidiol were noted in animals in the 1970s with the first human trials in 1980 (Cunha et al., 1980). A recent experiment in mice with seizures induced by pentylenetetrazole employed five different Cannabis extracts with equal CBD concentrations (Berman et al., 2018). Although all the extracts showed benefits compared to untreated controls, salient differences were observed in biochemical profiles of non-CBD cannabinoids, which, in turn, led to significant differences in numbers of mice developing tonic-clonic seizures (21.5–66.7%) and survival rates (85–100%), highlighting the relevance of these “minor” components. This study highlights the necessity of standardization in pharmaceutical development, and although it could be construed to support the single molecule therapeutic model (Bonn-Miller et al., 2018), it requires emphasis that complex botanicals can meet American FDA standards (Food and Drug Administration, 2015). Specifically, two Cannabis-based drugs have attained regulatory approval, Sativex^®^(nabiximols, US Adopted Name) in 30 countries, and Epidiolex^®^in the United States.

The question then arises: Can a Cannabis preparation or single molecule be too pure, thus reducing synergistic potential? Recent data support this as a distinct possibility. Anecdotal information from clinicians utilizing high-CBD Cannabis extracts to treat severe epilepsy, such as Dravet and Lennox-Gastaut syndromes, showed that their patients demonstrated notable improvement in seizure frequency (Goldstein, 2016; Russo, 2017; Sulak et al., 2017) with doses far lower than those reported in formal clinical trials of Epidiolex, a 97% pure CBD preparation with THC removed (Devinsky et al., 2016, 2017, 2018; Thiele et al., 2018). This observation was recently subjected to meta-analysis of 11 studies with 670 patients in aggregate (Pamplona et al., 2018). Those results showed that 71% of patients improved with CBD-predominant Cannabis extracts vs. 36% on purified CBD (*p* < 0.0001). The response rate at 50% improvement in seizure frequency was not statistically different in the two groups and both groups achieved seizure-free status in about 10% of patients. However, the mean daily doses were markedly divergent in the groups: 27.1 mg/kg/d for purified CBD vs. only 6.1 mg/kg/d. for CBD-rich Cannabis extracts, a dose only 22.5% of that for CBD alone. Furthermore, the incidence of mild and severe adverse events was demonstrably higher in purified CBD vs. high-CBD extract patients (*p* < 0.0001), a result that the authors attributed to the lower dose utilized, which was achieved in their opinion by the synergistic contributions of other entourage compounds. Such observations support the hypothesis of greater efficacy for Cannabis extracts combining multiple anticonvulsant components, such as CBD, THC, THCA, THCV, CBDV, linalool, and even caryophyllene (Lewis et al., 2018).

**FIGURE 2:**
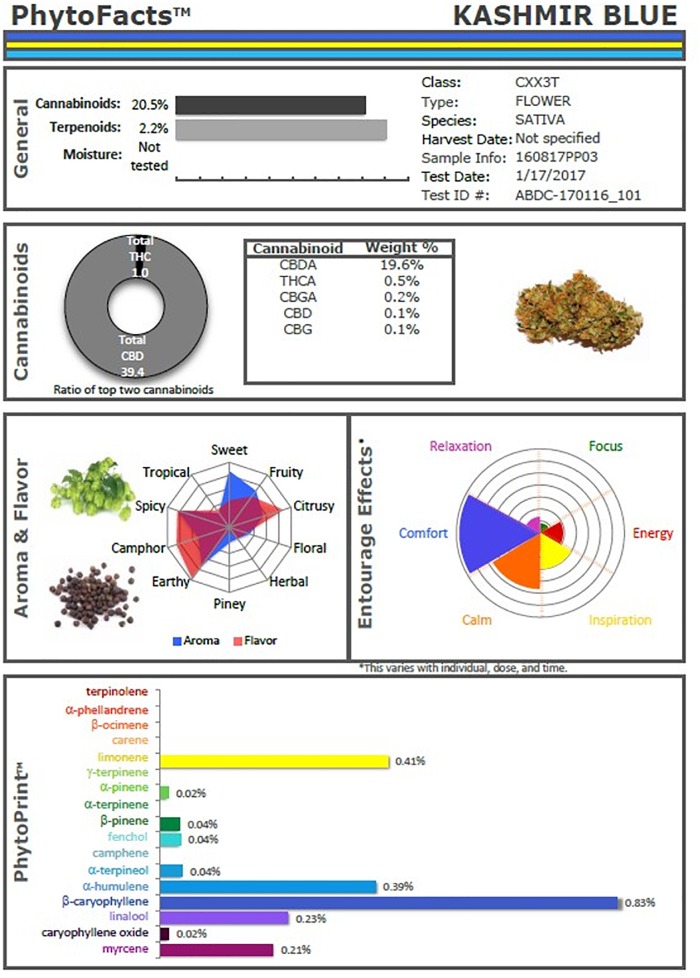
PhytoFacts^TM^ depiction of cannabinoid and terpenoid content of Caryodiol^TM^, aka “Kashmir Blue,” a Type III, cannabidiol-, and caryophyllene-predominant chemovar. See (Lewis et al., 2018) for details of PhytoFacts and conventional breeding methodology. Copyright© 2016 BHC Group, LLC. All rights reserved. Any unauthorized use of this document or the images or marks above may violate copyright, trademark, and other applicable laws.

These studies and others provide a firm foundation for Cannabis synergy, and support for botanical drug development vs. that of single components (Bonn-Miller et al., 2018), or production via fermentation methods in yeast or other micro-organisms. An example of the power of conventional selective breeding is illustrated (Figure 2), in the form of a Cannabis chemovar named Caryodiol^TM^ for its enhanced caryophyllene content (0.83%) as a CB_2_ agonist, along with highly favorable Type III THC:CBD ratio of 1:39.4. Such a preparation portends to be applicable to treatment of numerous clinical conditions including: pain, inflammation, fibrotic disorders, addiction, anxiety, depression, autoimmune diseases, dermatological conditions and cancer (Pacher and Mechoulam, 2011; Russo, 2011; Xi et al., 2011; Russo and Marcu, 2017; Lewis et al., 2018). Producing such a combination from microbial sources might require combinations of cannabinoids from multiple yeast species and, as a result, it would represent a combination product subject to a difficult regulatory path compared to Cannabis preparations from extracts of a single species (e.g., nabiximols) that has been accepted as a unitary formulation in 30 countries across the globe (Food and Drug Administration, 2015).

This article has briefly outlined recently technological attempts to “reinvent the phytocannabinoid wheel.” Cogent arguments would support that it can be done, but should it be done? The data supporting the existence of Cannabis synergy and the astounding plasticity of the Cannabis genome suggests a reality that obviates the need for alternative hosts, or even genetic engineering of *Cannabis sativa*, thus proving that, “The plant does it better.”

## Author Contributions

The author confirms being the sole contributor of this work and has approved it for publication.

## Conflict of Interest Statement

I am Director of Research for the International Cannabis and Cannabinoids Institute. We serve clients engaged in cannabis commerce.
